# Estimation of neuronal firing rate using Bayesian Adaptive Kernel Smoother (BAKS)

**DOI:** 10.1371/journal.pone.0206794

**Published:** 2018-11-21

**Authors:** Nur Ahmadi, Timothy G. Constandinou, Christos-Savvas Bouganis

**Affiliations:** 1 Centre for Bio-Inspired Technology, Institute of Biomedical Engineering, Imperial College London, London, United Kingdom; 2 Department of Electrical and Electronic Engineering, Imperial College London, London, United Kingdom; Indiana University, UNITED STATES

## Abstract

Neurons use sequences of action potentials (spikes) to convey information across neuronal networks. In neurophysiology experiments, information about external stimuli or behavioral tasks has been frequently characterized in term of neuronal firing rate. The firing rate is conventionally estimated by averaging spiking responses across multiple similar experiments (or trials). However, there exist a number of applications in neuroscience research that require firing rate to be estimated on a single trial basis. Estimating firing rate from a single trial is a challenging problem and current state-of-the-art methods do not perform well. To address this issue, we develop a new method for estimating firing rate based on a kernel smoothing technique that considers the bandwidth as a random variable with prior distribution that is adaptively updated under an empirical Bayesian framework. By carefully selecting the prior distribution together with Gaussian kernel function, an analytical expression can be achieved for the kernel bandwidth. We refer to the proposed method as Bayesian Adaptive Kernel Smoother (BAKS). We evaluate the performance of BAKS using synthetic spike train data generated by biologically plausible models: inhomogeneous Gamma (IG) and inhomogeneous inverse Gaussian (IIG). We also apply BAKS to real spike train data from non-human primate (NHP) motor and visual cortex. We benchmark the proposed method against established and previously reported methods. These include: optimized kernel smoother (OKS), variable kernel smoother (VKS), local polynomial fit (Locfit), and Bayesian adaptive regression splines (BARS). Results using both synthetic and real data demonstrate that the proposed method achieves better performance compared to competing methods. This suggests that the proposed method could be useful for understanding the encoding mechanism of neurons in cognitive-related tasks. The proposed method could also potentially improve the performance of brain-machine interface (BMI) decoder that relies on estimated firing rate as the input.

## Introduction

In neural systems, signaling and interneuronal communication can be observed through the characteristic of action potentials (or ‘spikes’). A sequence of spikes, known as a spike train, may encode information based on different schemes. Currently, there are two main hypotheses of neural coding schemes: *temporal* coding and *rate* coding. The temporal coding represents the information by the precise timing or occurrence of spikes. On the other hand, the rate coding represents the information by the rate or frequency at which a neuron “fires” spikes, also known as “firing rate”, and has been the most commonly used scheme to characterize the neuronal or network responses to external stimuli or behavioral tasks [1, 2]. The firing rate is typically estimated in offline analysis by averaging spiking responses across multiple repeated experiments known as trials. In practice, however, spiking responses may differ considerably even though the trial setting remains approximately the same. This is partly due to the inherent stochastic nature of neurons and the difference of cognitive states during the trials [3, 4]. Averaging out many variably similar trials can obscure the temporal dynamics, which may contain useful information, on each single trial. Furthermore, many research of interests require the firing rate to be estimated on single trial basis. For example, quantifying trial-to-trial variability of neuronal responses [5, 6], decoding task parameters in brain-machine interface (BMI) applications [3, 5], and measuring neuronal responses in cognitive-related tasks such as decision making, motor planning, learning and memory [7–9]. Therefore, it is essential to be able to accurately estimate firing rate based on single trials.

Estimating firing rate as a continuous-time function from a single trial is a challenging task since the underlying process provides only a sparse representation of the spiking data. A widely used method known as peri-stimulus time histogram (PSTH) results in a coarse estimate [10, 11]. To produce smooth estimate of firing rate, several methods have been proposed such as optimized kernel smoother (OKS) [12], variable kernel smoother (VKS) [12], local polynomial fit (Locfit) [13], and Bayesian adaptive regression splines (BARS) [14]. OKS and VKS employ kernel density estimation technique in which the accuracy of estimation is heavily impacted by the choice of the kernel bandwidth. Both methods automatically compute the bandwidth based on mean integrated squared error (MISE) minimization principle. However, in computing the bandwidth, these methods make assumption that spikes are generated by a Poisson process. Even though superimposed spike trains across many trials approximate a Poisson process, in the case of single trial, a spike train has been shown to depart from this assumption [15, 16]. Single trial spike train exhibits history-dependent properties such as refractory period and bursting, which cannot be modeled by Poisson process [15, 16]. The deviation from Poisson assumption can lead both OKS and VKS to exhibit poor performance under single trial cases. Locfit employs a generalized nonparametric regression technique where the firing rate is approximated by polynomial. The estimation accuracy of Locfit depends mostly on a smoothing parameter (bandwidth). How this bandwidth is selected along with the Poisson assumption of the spike train are the main caveats of this method. Like Locfit, BARS also employs a generalized regression technique, except that it estimates the firing rate using splines (several polynomials connected at some points or knots). The challenge of using a spline-based method is determining the number and location of the knots since these will significantly impact the estimation. To determine the optimal knot configurations (number and location), BARS utilizes a reversible-jump Markov chain Monte Carlo (MCMC) engine with Bayesian information criterion (BIC). The flexibility and powerfulness of BARS comes at a price of relatively high computational complexity [14, 17]. In addition, similarly to above-mentioned methods, BARS also assumes that spikes are generated from a Poisson process. It is intended to be used for firing rate estimation after pooling spikes across multiple trials [17].

Within this context, it is highly desirable to have a method for estimating neuronal firing rate from a single trial spike train, which features an adaptive capability and low computational complexity. An adaptive capability is crucial as spiking activity may change rapidly within single trial. The proposed method should be data-driven and able to accurately estimate the underlying spike dynamics. Low computational complexity is needed to perform the computation within a short time, and is necessary for real-time BMI applications. In addition, it is important to have a spike train model that mimics the spiking behavior encountered in real neural recording. Using certain assumption on the underlying rate function (i.e. the “ground truth” is known), single spike trains can be stochastically generated which can then be used to evaluate the performance of the firing rate estimation method.

In this paper, we propose a new method for estimation of firing rate that addresses the issues listed above. This method employs a kernel smoothing technique due to its simplicity. The key parameter, bandwidth, is considered as a random variable with a prior distribution and is adaptively determined under an empirical Bayesian framework. With the appropriate selection of kernel and prior distribution functions, an analytical expression of posterior distribution can be attained which reduces the computational complexity. We refer to this method as Bayesian Adaptive Kernel Smoother (BAKS). We evaluate BAKS with synthetic data generated from two biologically plausible models: inhomogeneous Gamma (IG) and inhomogeneous inverse Gaussian (IIG). BAKS is then tested with real neural data recorded from motor and visual cortex of non-human primate (NHP).

## Methods

In this section, we first introduce a kernel smoothing technique for estimating firing rate. We then describe our proposed method, BAKS, a new variant of kernel-based firing rate estimation method that incorporates an adaptive bandwidth. Lastly, we explain two models that we used to generate synthetic spike train data for evaluating the performance of the proposed method. The BAKS code and all the datasets that we synthesized (in Matlab) have been made publicly available through https://github.com/nurahmadi/BAKS.

### Kernel-based firing rate estimation

Let *t*_1_, *t*_2_, ⋯, *t*_*n*_ be a sequence of spike times (i.e. a spike train) which can be expressed mathematically as

$$\begin{array}{c} \rho \left(t\right)=\sum_{i=1}^{n}\delta \left(t-t_{i}\right) \end{array}$$ (1)

where *δ*(*t*) is Dirac function and *n* is the total number of spikes. The underlying rate function also known as firing rate, λ(*t*), can be estimated by using kernel smoothing, a method which convolves the spike train with a kernel function *K*(*t*) as follows,

$$\begin{array}{c} \hat{\lambda }\left(t\right)=\int_{-\infty }^{\infty }K\left(\tau \right)\rho \left(t-\tau \right)d\tau \end{array}$$ (2)

Eq (2) can also be represented as the sum over kernel functions centered at spike times *t*_*i*_,

$$\begin{array}{c} \hat{\lambda }\left(t\right)=\sum_{i=1}^{n}K\left(t-t_{i}\right) \end{array}$$ (3)

The effectiveness of kernel smoothing technique depends on the choice of a kernel function and the selection of a smoothing parameter (i.e. bandwidth). A kernel function is required to be a non-negative, normalized to a unit area, having a zero first moment and a finite variance [5, 12]. Examples of kernel functions that have been widely used are Gaussian and Epanechnikov [18, 19]. In many occasions, the kernel bandwidth is set fixed over the whole observation interval. A significant amount of literature has been reported in the field of statistics on selecting the proper value for this fixed bandwidth [20–24]. Even though a near-optimal fixed bandwidth selection (e.g. [12, 25, 26]) may yield a better estimate compared to an arbitrary choice, it may still suffer from simultaneously under- and over-smoothing depending on the underlying spike dynamics. A rapid change in spiking activity is sometimes encountered in neural responses and is of interest to neuroscientists. Thus, it is highly desirable to find optimal adaptive bandwidth selection method that can adaptively grasp the slow and rapid changes of firing rate.

### Bayesian Adaptive Kernel Smoother (BAKS)

BAKS employs a kernel smoothing technique and incorporates an adaptive bandwidth at the estimation points, meaning the bandwidth at which firing rate is being estimated can adapt to the dynamics of the underlying process. In so doing, BAKS considers the bandwidth as a random variable with prior distribution and adaptively updates the posterior bandwidth given spiking data using an empirical Bayesian framework.

#### Selection of kernel function

There are several choices of kernel functions that can be utilized for firing rate estimation. Gaussian and Epanechnikov are among the popular kernel functions in statistical research [18, 19]. In term of minimizing asymptotic mean integrated squared error (AMISE), Gaussian kernel is slightly less efficient than Epanechnikov kernel [18]. However, Gaussian offers more interesting properties, e.g. the availability of several types of conjugate prior distributions. This conjugate property enables an analytical expression of posterior distribution which simplifies the computation and avoids using a numerical approximation technique. Due to this mathematical convenience, in our proposed method, we select a Gaussian kernel with adaptive bandwidth which can be expressed as,

$$\begin{array}{c} K\left(t\right)=\frac{1}{\sqrt{2\pi }h\left(t\right)}\exp\{-\frac{t^{2}}{2h{\left(t\right)}^{2}}\} \end{array}$$ (4)

where *h*(*t*) is the adaptive bandwidth. The bandwidth should be small (large) at the region of high (low) spike density.

#### Selection of prior distribution

We select a prior distribution of bandwidth that incorporates prior belief about spiking data (i.e. informative prior) and also leads to an analytical expression of posterior distribution. The informative prior is especially useful when the number of spiking data is relatively small as this prior can give more weight than the likelihood function, while the analytical expression is crucial to simplifying the computation and avoiding a numerical approximation technique (e.g. Markov chain Monte Carlo). In Eq (4), the Gaussian kernel uses parameter bandwidth (i.e. standard deviation in statistical literature) that describes how spread the observed data are around the mean. We can also represent the parameter in term of precision (inverse of square bandwidth) that describes how concentrated the observed data are around the mean. In computing firing rate estimation, the means of Gaussian kernel are set to the spike times. These spike times can be represented as sum of the interspike intervals (ISIs) that can be conveniently modeled by Gamma distribution [15, 27–30]. Since sum of independent Gamma random variables follows Gamma distribution [31], the spike times can also be represented as Gamma distribution. Hence, we propose a Gamma prior distribution on the precision parameter *σ*(*t*), where *σ*(*t*) = 1/*h*(*t*)^2^. As Gamma distribution is a conjugate prior for Gaussian distribution with precision parameter [32], the choice of Gamma prior distribution results in an analytical expression of the posterior distribution. This Gamma prior distribution is given by

$$\begin{array}{c} \pi \left(\sigma \left(t\right)\right)=\frac{\sigma {\left(t\right)}^{\alpha -1}}{\Gamma \left(\alpha \right)\beta^{\alpha }}\exp\{-\frac{\sigma (t)}{\beta }\},\;\sigma >0 \end{array}$$ (5)

where *α* > 0 is the shape parameter, *β* > 0 is the scale parameter, and Γ(*α*) is Gamma function. By the change-of-variable formula and transformation technique, we can express the prior distribution *π*(*σ*(*t*)) as a function of *h*(*t*):

$$\begin{array}{c} \begin{array}{cc} \pi (h(t)) & =\pi \left(\sigma \left(t\right)\right)\times |\frac{d\sigma (t)}{dh(t)}|\\ & =\frac{2h{\left(t\right)}^{-2\alpha -1}}{\Gamma \left(\alpha \right)\beta^{\alpha }}\exp\{-\frac{1}{\beta h{\left(t\right)}^{2}}\} \end{array} \end{array}$$ (6)

#### BAKS modeling and inference

Under Bayesian framework, the statistical inference depends on both likelihood function and prior distribution. The likelihood function is the probability density function of the observed spike train *ρ*(*t*) viewed as a function of the unknown bandwidth parameter *h*(*t*), which can be approximated by

$$\begin{array}{c} \begin{array}{cc} \hat{f}\left(\rho \left(t\right)|h\left(t\right)\right) & =\frac{1}{n}\sum_{i=1}^{n}K_{h(t)}\left(t-t_{i}\right)\\ & =\frac{1}{n}\sum_{i=1}^{n}\frac{1}{\sqrt{2\pi }h\left(t\right)}\exp\{-\frac{{\left(t-t_{i}\right)}^{2}}{2h{\left(t\right)}^{2}}\} \end{array} \end{array}$$ (7)

where *K*_*h*(*t*)_(*t* − *t*_*i*_) is Gaussian kernel as defined in Eq (4) with adaptive bandwidth and centered at spike event time *t*_*i*_.

Using Bayes’ theorem, the posterior distribution of kernel bandwidth, *π*(*h*(*t*)|*ρ*(*t*)), can be computed by

$$\begin{array}{c} \pi \left(h\left(t\right)|\rho \left(t\right)\right)=\frac{\hat{f}\left(\rho \left(t\right)|h\left(t\right)\right)\pi \left(h\left(t\right)\right)}{\int \hat{f}\left(\rho \left(t\right)|h\left(t\right)\right)\pi \left(h\left(t\right)\right)dh\left(t\right)} \end{array}$$ (8)

Computing the denominator of Eq (8), also called marginal density, can be problematic if there is no analytical solution of its integral expression since an approximation method will be required instead. The choice of prior distribution in Eq (6) coupled with Gaussian kernel in Eq (7) leads to an analytical expression for the denominator in Eq (8) as follows,

$$\begin{array}{c} \pi \left(h\left(t\right)|\rho \left(t\right)\right)=\frac{\sum_{i=1}^{n}h{\left(t\right)}^{-2\alpha -2}\exp\{-\frac{1}{h{\left(t\right)}^{2}}[\frac{{\left(t-t_{i}\right)}^{2}}{2}+\frac{1}{\beta }]\}}{\frac{1}{2}\Gamma \left(\alpha +\frac{1}{2}\right)\sum_{i=1}^{n}{\left[\frac{{\left(t-t_{i}\right)}^{2}}{2}+\frac{1}{\beta }\right]}^{\left(-\alpha -\frac{1}{2}\right)}} \end{array}$$ (9)

The adaptive bandwidth can then be estimated under squared error loss function by using the posterior mean formulated as

$$\begin{array}{c} \hat{h}\left(t\right)=\int h\left(t\right)\pi \left(h\left(t\right)|\rho \left(t\right)\right)dh\left(t\right) \end{array}$$ (10)

The closed-form expression of Eq (10) is given by

$$\begin{array}{c} \hat{h}\left(t\right)=\frac{\Gamma \left(\alpha \right)\sum_{i=1}^{n}{\left[\frac{{\left(t-t_{i}\right)}^{2}}{2}+\frac{1}{\beta }\right]}^{-\alpha }}{\Gamma \left(\alpha +\frac{1}{2}\right)\sum_{i=1}^{n}{\left[\frac{{\left(t-t_{i}\right)}^{2}}{2}+\frac{1}{\beta }\right]}^{-\alpha -\frac{1}{2}}} \end{array}$$ (11)

Eqs (9)–(11) define what is called a mixture of *t*-distributions in statistical literature. By substituting the adaptive bandwidth in Eq (11) into Eq (3), we can compute firing rate estimation using the following formula:

$$\begin{array}{c} \hat{\lambda }\left(t\right)=\sum_{i=1}^{n}\frac{1}{\sqrt{2\pi }\hat{h}\left(t\right)}\exp\{-\frac{{\left(t-t_{i}\right)}^{2}}{2\hat{h}{\left(t\right)}^{2}}\} \end{array}$$ (12)

**Fig 1:**
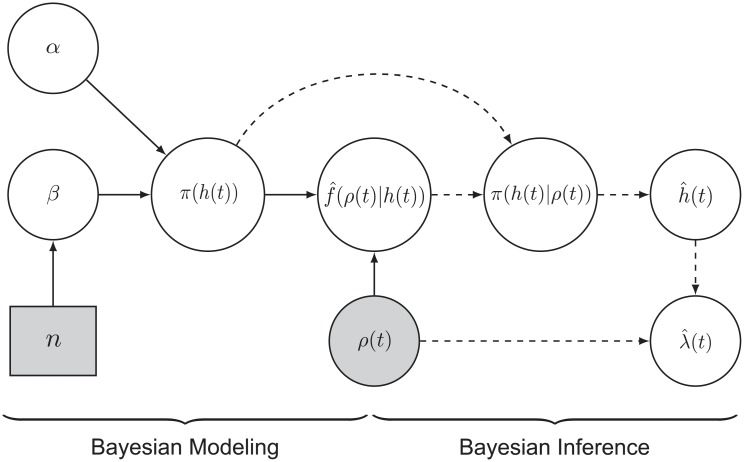
Graphical representation of BAKS. The various blocks describe the different components as follows: Circular blocks denote continuous variables, and rectangle blocks denote discrete variables. Shaded blocks represent observed variables, whereas white blocks represent hidden variables. Solid and dashed arrow indicate Bayesian modeling and inference phase, respectively.

BAKS is graphically illustrated in Fig 1. The derivation of the closed-form expressions of bandwidth posterior distribution and the adaptive bandwidth estimate are given in Appendix 1 and Appendix 2, respectively.

#### Parameter setting of prior distribution

The effectiveness of firing rate estimation using BAKS depends on the adaptive bandwidth estimate, which is influenced by the setting of parameter shape (*α*) and scale (*β*) of the prior distribution. According to prior distribution of bandwidth in Eq (6), we can calculate its mean and variance as follows,

$$\begin{array}{c} \begin{array}{cc} E[h(t)] & =\int h(t)\pi (h(t))dh(t)\\ & =\frac{\Gamma (\alpha -\frac{1}{2})}{\Gamma \left(\alpha \right)\beta^{\frac{1}{2}}} \end{array} \end{array}$$ (13)

$$\begin{array}{c} \begin{array}{cc} V[h(t)] & =\int {\left(h\left(t\right)-\mu \right)}^{2}\pi \left(h\left(t\right)\right)dh\left(t\right),\;\mu =E\left[h\left(t\right)\right]\\ & =\frac{\Gamma \left(\alpha \right)\Gamma \left(\alpha -1\right)-\Gamma {\left(\alpha -\frac{1}{2}\right)}^{2}}{\Gamma {\left(\alpha \right)}^{2}\beta } \end{array} \end{array}$$ (14)

where E[h(t)]andV[h(t)] are the mean and variance of *π*(*h*(*t*)), respectively. It can be observed from both Eqs (13) and (14) that the mean and variance are inversely proportional to the value of parameter *β*. As the number of spike events (*n*) increases, the bandwidth decreases. Hence, to obtain consistency of the estimation, the value of *β* is set to be a function proportional to the number of spike events (*n*). Here, we propose *β* = *n*^4/5^ in accordance with MISE convergence rate of Gaussian kernel [33].

To yield accurate estimate of firing rate, we tune parameter *α* by minimizing mean integrated squared error (MISE). Since *h*(*t*) > 0, the numerator of Eqs (13) and (14) must be greater than zero, which in turn requires *α* > 1. The value of parameter *α* is tuned such that it minimizes MISE function given fixed value of *β* = *n*^4/5^. This tuning process is mathematically expressed as follows,

$$\begin{array}{c} \alpha_{t}=\arg\min_{\alpha }\left\{\text{MISE}\left(\alpha \right)\right\} \end{array}$$ (15)

MISE is a common metric used in evaluating goodness-of-fit of a density estimation and has been used in previous studies of firing rate estimation [5, 12, 34]. It is computed between the estimated firing rate ($\hat{\lambda }\left(t\right)$) and the true underlying rate function (λ(*t*)) with the following formula,

$$\begin{array}{c} \begin{array}{cc} \text{MISE} & =\int E{\left[\hat{\lambda }\left(t\right)-\lambda \left(t\right)\right]}^{2}dt\\ & \approx \Delta t\sum E{\left[\hat{\lambda }\left(t\right)-\lambda \left(t\right)\right]}^{2} \end{array} \end{array}$$ (16)

where E denotes the expectation with respect to stochastic process of spike generation model and the integration/summation is performed over observation interval.

The tuning of parameter *α* was performed using synthetic spike train data generated by biologically plausible models –inhomogeneous Gamma (IG) model and inhomogeneous inverse Gaussian (IIG) model– which are described in the subsequent section. Parameter *α* value that gives the smallest MISE during this tuning process was then fixed and used for firing rate estimation during performance evaluation (testing) phase of our proposed method. The testing phase was performed using distinct synthetic datasets (i.e. different than that of during tuning phase) and real neural datasets.

### Spike train generation model

It is essential to have a model for generating spike trains in order to tune the parameter of BAKS and to evaluate its performance. A spike train can be modeled by using a point process; a stochastic process that describes localized events or points in real (e.g. time, space) domain. The most commonly used class of point processes to model a spike train is inhomogeneous Poisson (IP) process. [3, 5, 12, 15, 25]. In this class, spike counts (increments) within any intervals vary across time (i.e. nonstationary). The spike counts depend on integral value of the firing intensity function over interval in question but do not depend on the past spike times (i.e. remain independent). As a consequence, IP process still possesses memoryless property; a spike occurring at a particular time does not depend on the past spiking activities [35]. It has been shown that IP process can be used to approximate the behavior of neural spikes when the spikes are superimposed across trials [12, 16]. However, a Poisson process cannot model the history-dependent properties (e.g. refractory period and bursting) of neural spike trains in the case of single trial. Hence, another class of point process is required to model single trial spike train.

*Renewal process* is an alternative class of point process that can describe the history-dependent property of spike times by assuming that a spike fired at any point in time depending only on the last spike, not the spikes before it. In the renewal process, spike times are no longer independent, but it is their inter-spike intervals (ISIs) that remain independent. Spike trains can be conveniently represented by their ISIs drawn from a certain distribution. Two classes of distribution which have often been used to model non-Poisson spike train are Gamma and inverse Gaussian distributions [6, 15, 27–30, 36]. The former is then called inhomogeneous Gamma (IG) model while the latter is called inhomogeneous inverse Gaussian (IIG) model.

#### Inhomogeneous Gamma (IG) model

The Inhomogeneous Gamma (IG) model generalizes a Poisson model by allowing more flexible ISI distribution that is controlled by shape parameter (*γ*) [15, 27–30]. The gamma probability density of the ISI is defined as

$$\begin{array}{c} f_{\tau }\left(\tau \right)=\frac{1}{\Gamma \left(\gamma \right)\theta^{\gamma }}\tau^{\gamma -1}\exp\{-\frac{\tau }{\theta }\} \end{array}$$ (17)

where *τ* > 0 denotes the ISI, *γ* > 0 represents the shape parameter, *θ* is the scale parameter, and Γ(*γ*) is the Gamma function. As shown in Eq (17), when *γ* = 1, the ISI follows an exponential distribution as in a Poisson process. If *γ* < 1, the probability density value declines faster than exponential, which means the ISI tends to become smaller. This can be used to describe neuron’s rapid firing (bursting) phenomena. If *γ* > 1, the probability density value will increase from zero up to a certain peak value and then decrease again to zero. This indicates the refractory period property where a neuron is less likely to fire again immediately after it fires a spike.

To generate a spike train from such a model, we employ time-rescaling theorem which transforms the original spike times into new rescaled spike times where the ISIs are independently and identically drawn from fixed distribution (e.g. Gamma) [29]. In the IG model, this transformation is defined as integration of underlying rate function, λ(*t*), on the interval (0, *t*] and multiplied by the shape parameter (*γ*). This transformation is formulated as

$$\begin{array}{c} \Lambda \left(t\right)=\gamma \int_{0}^{t}\lambda \left(u\right)du \end{array}$$ (18)

Since λ(*t*) is a non-negative function, Λ(*t*) is a monotonically increasing and one-to-one function and usually called integrated intensity function. Thus, we can obtain the original spike times from *i.i.d*. ISI samples (*τ*) by performing the inverse of time-rescaling transform as follows,

$$\begin{array}{c} \tau_{i}\equiv \Lambda_{t_{i-1}}\left(t_{i}\right)=\Lambda \left(t_{i}\right)-\Lambda \left(t_{i-1}\right)=\gamma \int_{t_{i-1}}^{t_{i}}\lambda \left(u\right)du \end{array}$$ (19)

$$\begin{array}{c} t_{i}=\Lambda_{t_{i-1}}^{-1}\left(\tau_{i}\right) \end{array}$$ (20)

where 0 < *t*_1_ < *t*_2_ <, ⋯, < *t*_*n*_ ≤ *T* represent the spike times within interval (0, *T*] and $\Lambda_{t_{i-1}}^{-1}\left(t\right)$ is the inverse of time-rescaling transform.

#### Inhomogeneous inverse Gaussian (IIG) model

It has been suggested that inhomogeneous inverse Gaussian (IIG) model is more biologically plausible to describe the characteristic of neural spike train than IG model [15, 37]. The IIG model uses inverse Gaussian distribution for the ISI and has been applied in multiple studies [38, 39]. The inverse Gaussian ISI distribution used in this model is given by

$$\begin{array}{c} f_{\tau }\left(\tau \right)={\left[\frac{\gamma }{2\pi \tau^{3}}\right]}^{1/2}\text{exp}\left\{-\frac{\gamma {\left(\tau -\mu \right)}^{2}}{2\mu^{2}\tau }\right\} \end{array}$$ (21)

where *τ* > 0 denotes the ISI, *γ* is the shape parameter, and *μ* is the location parameter. In the IIG model, the value of ISI density is zero at the origin, then increases to certain peak value, and decreases again to zero. How quick the density value rises or falls is controlled by the shape parameter (*γ*), which demonstrates the flexibility of IIG model in describing different neural spike characteristics such as rapid bursting and refractory period. The smaller (larger) value of *γ*, neuron tends to fire more bursty (regularly).

To generate a spike train using the IIG model, we employ a similar procedure as that of IG model, with the exception that the time-rescaling transform here is expressed as

$$\begin{array}{c} \Lambda \left(t\right)=\int_{0}^{t}\lambda \left(u\right)du \end{array}$$ (22)

where the interval between subsequent spike times (ISIs) in the rescaled domain are drawn from *i.i.d*. inverse Gaussian distribution. The spike times in the original domain can be computed by using the inverse of time-rescaling transform as shown in Eq (20).

## Results

### Synthetic datasets

In our synthetic data, the spike trains were generated from IG and IIG models with different underlying rate functions that represent non-stationary processes usually encountered in empirical datasets. These rate functions include (1) a continuous process with changing (heterogeneous) frequency, (2) a continuous process with oscillatory (homogeneous) frequency, and (3) a discontinuous process with sudden rate changes. In this study, the first, second, and third rate functions are referred to as ‘chirp’, ‘sine’, and ‘sawtooth’ rate functions, and are mathematically expressed as,

$$\begin{array}{c} \lambda_{c}\left(t\right)=\eta +A\sin\left(2\pi ft^{2}+\phi \right) \end{array}$$ (23)

$$\begin{array}{c} \lambda_{s}\left(t\right)=\eta +A\sin\left(2\pi ft+\phi \right) \end{array}$$ (24)

$$\begin{array}{c} \lambda_{st}\left(t\right)=\eta +\frac{2A}{\pi }\arctan\left(\cot\left(\pi ft+\phi \right)\right) \end{array}$$ (25)

where *η* indicates the base or average number of spikes per second and *A* denotes the intensity (or amplitude) which controls the dynamic range of the rate function. Parameter *f* is frequency whereas *ϕ* is phase. The chirp, sine and sawtooth rate functions are represented by λ_*c*_(*t*), λ_*s*_(*t*) and λ_*st*_(*t*), respectively.

**Fig 2:**
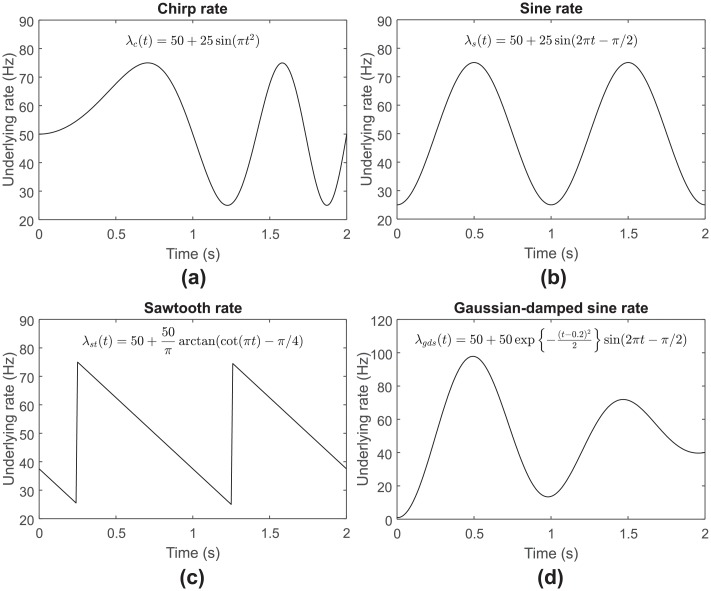
Illustration of the underlying rate functions used in this study. (a) Chirp rate function with *η* = 50, *A* = 25, *f*_*c*_ = 0.5, and *ϕ* = 0. (b) Sine rate function with *η* = 50, *A* = 25, *f*_*s*_ = 1, and *ϕ* = −*π*/2. (c) Sawtooth rate function with *η* = 50, *A* = 25, *f*_*st*_ = 1, and *ϕ* = −*π*/4. (d) Gaussian-damped sinusoidal rate function with *η* = 50, *A* = 1, *t*_0_ = 0.2, *σ* = 1, *f*_*gds*_ = 0.5, and *ϕ* = −*π*/2.

The chirp rate function used in this study is similar to that of Rao and Teh [40], whereas the sine and sawtooth processes are similar to that of Shimazaki and Shinomoto [12] but with different intensity, frequency, and spike train model. Examples of chirp, sine, and sawtooth rate functions with certain parameter settings are illustrated in Fig 2a, 2b and 2c, respectively.

During parameter tuning of BAKS (tuning phase), we set *η* to 50 spikes/s and *A* to 25 spikes/s (corresponds to dynamic intensity range between 25 and 75 spikes/s) as in [28]. These intensity values are referred to as medium intensity. This setting is reasonable as in practice cortical neurons do not often fire more than 100 spikes/s [41]. The frequency of chirp (*f*_*c*_), sine (*f*_*s*_) and sawtooth (*f*_*st*_) rate functions were set to 0.5, 1 and 1, respectively. These frequency values are referred to as medium frequency. We generated randomly 100 single spike trains (duration of 2s) from two models (IG and IIG) and three rate functions (chirp, sine, and sawtooth). In IG model, the shape parameter (*γ*) that determines the regularity of firing rate was set to 4, while the scale parameter (*θ*) was set to 1. This setting is comparable to real neural spiking data and also used in [27, 36]. Analogously, the shape (*γ*) and location (*μ*) parameters in IIG model were respectively set to 4 and 1. The parameter value (*α*) of prior distribution in BAKS was tuned by minimizing the average MISE from two models and three rate functions mentioned above. The same procedure was performed to tune the smoothing parameter value of Locfit.

For evaluating the performance of BAKS (testing phase), we generated 5 datasets each containing 100 repetitions from both IG and IIG models with the same and different setting from that of parameter tuning phase. Dataset testing 1 was generated using the same setting as in the tuning phase. Dataset testing 1 was used to evaluate BAKS when the underlying rate functions are the same but their stochastic spiking data realizations are different. Dataset testing 2 was used to evaluate BAKS when the frequency and intensity of the underlying rate functions are different. The frequency of chirp, sine and sawtooth rate functions were respectively set to low (0.25, 0.5 and 0.5) and high (0.75, 1.5 and 1.5) while keeping the intensity same as that of tuning phase. Then, the dynamic intensity range of these three rate functions were set to low (2–20) and high (25–175) while keeping the frequency same as that of tuning phase. In dataset testing 3, the model setting was equal to during the tuning phase except that the parameter shape (*γ*) was varied between 1 and 10 with an increment of 1. Dataset testing 4 was used to assess the performance of BAKS under multiple trials (*tr* = {5, 10, 20, 30}). These multi trial spike trains were obtained by superimposing spike trains from a number of trial (5, 10, 20 or 30) into single spike trains with the same setting as of tuning phase. In dataset testing 5, spike trains were generated using Gaussian-damped sinusoidal rate function with variable intensity and frequency. The idea of using this rate is taken from Mazurek’s work [41]. Mathematically, the Gaussian damped sinusoidal rate function is given by

$$\begin{array}{c} \lambda_{gds}\left(t\right)=\eta +\eta A\exp\{-\frac{{\left(t-t_{0}\right)}^{2}}{2\sigma^{2}}\}\sin\left(2\pi ft+\phi \right) \end{array}$$ (26)

During the generation of dataset testing 5, the frequency (*f*_*gds*_) was set to low (0.5), medium (1.5) and high (2) while keeping the dynamic intensity range to medium (0 to 100) which corresponds to *η* = 50 and *A* = 1; the dynamic intensity range was varied between low (0 to 20), medium (0 to 100) and high (0 to 200) while setting *f*_*gds*_ = 1. An example of Gaussian-damped sinusoidal rate function is illustrated in Fig 2d. The summary of model settings for generating the synthetic datasets used in tuning and testing phase is shown in Table 1.

**Table 1:** Model setting for synthetic dataset generation.

Phase	Rate function	Intensity	Frequency	Parameter shape (*γ*)	Number of trial (*tr*)
Tuning	{Chirp, sine, sawtooth}	Medium	Medium	4	1
Testing 1	{Chirp, sine, sawtooth}	Medium	Medium	4	1
Testing 2	{Chirp, sine, sawtooth}	{Low, high}	{Low, high}	4	1
Testing 3	{Chirp, sine, sawtooth}	Medium	Medium	{1, 2, ⋯, 10}	1
Testing 4	{Chirp, sine, sawtooth}	Medium	Medium	4	{5, 10, 20, 30}
Testing 5	{Gaussian-damped sine}	{Low, medium, high}	{Low, medium, high}	4	1

Tuning: medium intensity (*η* = 50, *A* = 25), medium frequency (*f*_*c*_ = 0.5, *f*_*s*_ = 1, *f*_*st*_ = 1)

Testing 1: medium intensity (*η* = 50, *A* = 25), medium frequency (*f*_*c*_ = 0.5, *f*_*s*_ = 1, *f*_*st*_ = 1)

Testing 2: low intensity (*η* = 11, *A* = 9), high intensity (*η* = 100, *A* = 75), low frequency (*f*_*c*_ = 0.25, *f*_*s*_ = 0.5, *f*_*s*_*t* = 0.5), high frequency (*f*_*c*_ = 0.75, *f*_*s*_ = 1.5, *f*_*st*_ = 1.5)

Testing 3: medium intensity (*η* = 50, *A* = 25), medium frequency (*f*_*c*_ = 0.5, *f*_*s*_ = 1, *f*_*st*_ = 1)

Testing 4: medium intensity (*η* = 50, *A* = 25), medium frequency (*f*_*c*_ = 0.5, *f*_*s*_ = 1, *f*_*st*_ = 1)

Testing 5: low intensity (*η* = 10, *A* = 1), medium intensity (*η* = 50, *A* = 1), high intensity (*η* = 100, *A* = 1), low frequency (*f*_*gds*_ = 0.5), medium frequency (*f*_*gds*_ = 1.5), high frequency (*f*_*gds*_ = 2)

### Tuning the shape parameter (*α*) of prior distribution

We investigated the choice of parameter value that yielded the most accurate firing rate estimates measured by MISE metric. The tuned parameter (*α*_*t*_) obtained from the tuning phase was then used as parameter value for our proposed method during testing phase. Spike trains used during tuning phase were stochastically generated from two models (IG and IIG) and three different underlying rate functions (chirp, sine, sawtooth) as described in previous section. We performed firing rate estimation over tuning dataset using parameter *α* varying from 1 to 10 with an increment of 0.5. This estimation was carried out while keeping parameter *β* fixed to *n*^4/5^, where *n* represents total number of spikes within observed duration.

**Fig 3:**
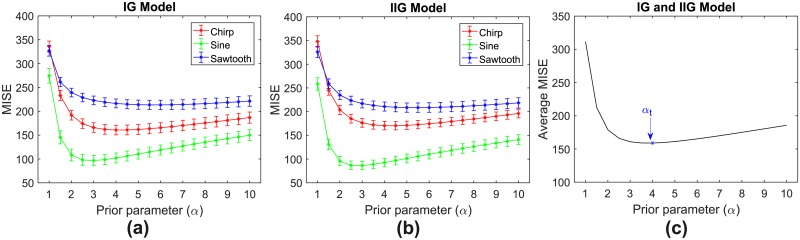
MISE comparison under different values of prior parameter (*α*). (a) MISE values (star marks) and their 95% confidence intervals (vertical bars) for IG model. (b) MISE values (star marks) and their 95% confidence intervals (vertical bars) for IIG model. (c) Average MISE values across 2 models and 3 rate functions. We set *α*_*t*_ = 4 as it yields the smallest average MISE.

The performance of estimation was quantified in term of MISE, mean of integrated squared error between the underlying and estimated firing rates over the observed duration (2s). From 100 repetitions and 19 variation of parameter values (*α* = {1, 1.5, 2, ⋯, 10}), we computed the MISE values along with their 95% confidence intervals from these 6 scenarios (2 models and 3 rate functions). The MISE values and their confidence intervals for IG and IIG models are shown in Fig 3a and 3b. In IG model, *α* values that resulted in smallest MISE for chirp, sine and sawtooth rate functions are 4, 3 and 6, respectively. In IIG model, *α* values associated with the smallest MISE for chirp, sine and sawtooth rate functions are 4.5, 3 and 5.5, respectively. We tuned parameter *α* by computing the average MISE values across 6 scenarios. The value of *α* that corresponds to the smallest average MISE is referred to as the tuned parameter (*α*_*t*_). According to the results as shown in Fig 3c, we set *α*_*t*_ = 4 and used this value during the testing phase.

### Comparison with established methods

We evaluated and compared the performance of the proposed method (BAKS) with established methods which include optimized kernel smoother (OKS) [12], variable kernel smoother (VKS) [12], local polynomial fit (Locfit) [13] and Bayesian adaptive regression splines (BARS) [14]. The performances were quantified using MISE as expressed in Eq (16). We did not include the the histogram (PSTH) method in the comparison as this cannot produce a smooth estimate under single-trial case. Shimazaki and Shinomoto demonstrated that even if the number of trials is increased, the performance of PSTH is far outperformed by OKS, VKS, Locfit, and BARS [12].

The two kernel smoothing methods used in the presented work, OKS and VKS, were developed by Shimazaki and Shinomoto [12]. In OKS, the bandwidth is fixed for the whole duration and automatically selected based on global MISE minimization principle. Unlike OKS, VKS employs a variable bandwidth and this bandwidth is automatically determined by minimizing local MISE function. Thus, both OKS and VKS do not require manual user intervention in selecting optimal bandwidth parameter. The Matlab codes for OKS and VKS can be obtained from the author’s website (http://www.neuralengine.org/res/kernel.html).

Locfit is part of Chronux analysis software that at the time of this study can be downloaded from http://chronux.org/. This method estimates the firing rate by maximizing local log-likelihood where the log-density function is approximated by a local polynomial. Locfit has some parameters such as degree of polynomial, weight function, and bandwidth. However, it is the bandwidth that is considered as the most crucial parameter affecting the accuracy of estimation [13]. We used nearest neighbor bandwidth so that the local neighborhood always contains sufficient data (spikes). This can reduce data sparsity problem that may arise in real neural data. The nearest neighbor bandwidth parameter was determined by a tuning process similar to that of BAKS, while the parameter of degree of polynomial and weight function were set to the default values which are two and tricube (‘tcub’), respectively. We performed MISE comparison from three underlying rate processes (chirp, sine, and sawtooth) for nearest neighbor bandwidth between 0.2 to 0.8 with increment 0.1. We set the bandwidth parameter value to 0.4 since the average MISE associated with this value was the smallest among others. The value 0.4 means that Locfit uses 40% of the total data in each estimation point.

BARS estimates the firing rate by using a cubic spline basis function with free parameters on the number and location of knots [14]. The optimal knot configuration is determined by a fully Bayesian approach with a reversible-jump Markov chain Monte Carlo (MCMC) engine and locality heuristic procedure. BARS takes spike counts within bin intervals (histogram) centered on estimation times of interest. In our study, we used Matlab implementation of BARS which is available at http://lib.stat.cmu.edu/~kass/bars/bars.html. We used a Poisson prior distribution on the number of knots and set the sample iterations to 5000 and burn-in samples to 500. We used spike counts within 10ms bin intervals as the input and mean of fitted function as the output estimate.

**Fig 4:**
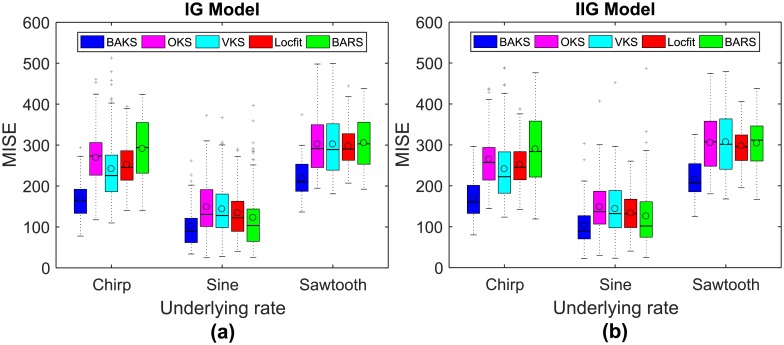
MISE comparison of BAKS with other methods. (a) MISE comparison for three rate functions (chirp, sine, and sawtooth) from IG model. (b) MISE comparison for three rate functions (chirp, sine, and sawtooth) from IIG model. In each boxplot, black lines show the medians; black circles indicate the means; colored solid boxes represent interquartile ranges; whiskers extend 1.5× from upper and lower quartiles; gray crosses represent outliers.

We performed 100 repetitions of single-trial firing rate estimation using dataset testing 1. MISE comparison of all the methods for three underlying rate functions and two models is plotted as a boxplot (Fig 4). In all 6 scenarios, the medians, means, and interquartile ranges of MISE computed by BAKS are smallest among other competing methods. As shown in Fig 4a for IG model and Fig 4b for IIG model, BAKS (blue boxplot) yields significantly lower MISE compared to the other methods. This demonstrates the effectiveness of BAKS which features an adaptive bandwidth in estimating the underlying rate from single trial spike train.

**Fig 5:**
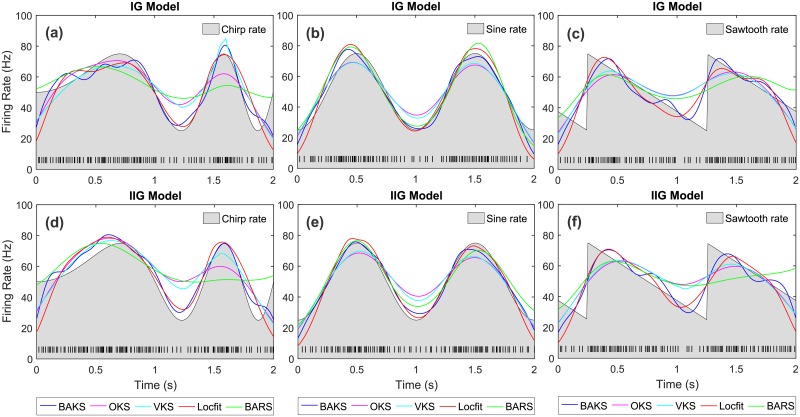
Comparison of firing rate estimates across all methods. (a)-(c) Firing rate estimates from IG model with chirp, sine, and sawtooth rate functions, respectively. (d)-(f) Firing rate estimates from IIG model with chirp, sine, and sawtooth rate functions, respectively. Black lines with gray-shaded regions indicate the underlying rate functions. Black raster in the bottom of each plot represents the spike train generated from the associated model and underlying rate function.

Examples of the underlying rate functions and the estimated firing rates from all methods are given in Fig 5. In this figure, BAKS (blue line) shows qualitatively good firing rate estimates across 6 scenarios.

### Comparison under different values of intensity and frequency

**Fig 6:**
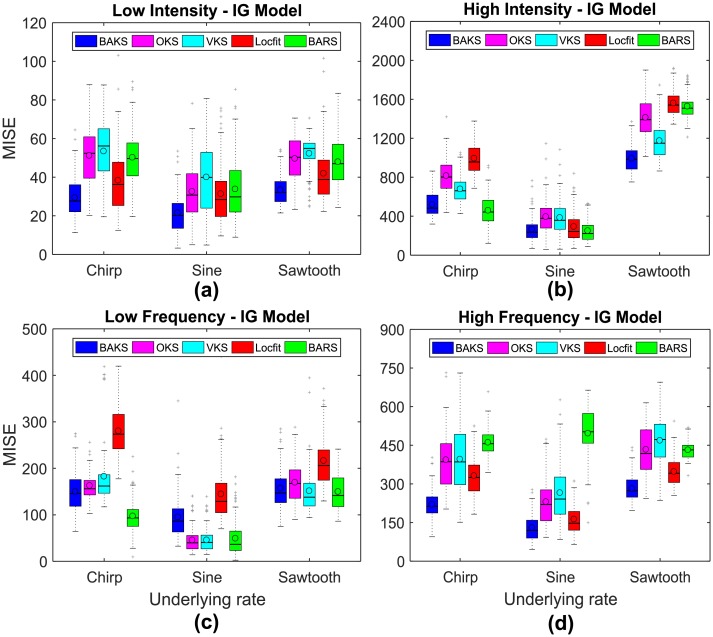
MISE comparison under different values of intensity and frequency for IG model. (a) MISE comparison for the case of low intensity. (b) MISE comparison for the case of high intensity. (c) MISE comparison for the case of low frequency. (d) MISE comparison for the case of high frequency. In each boxplot, black lines show the medians; black circles indicate the means; colored solid boxes represent interquartile ranges; whiskers extend 1.5× from upper and lower quartiles; gray crosses represent outliers.

We studied the effect of different intensity and frequency values of the underlying rate functions to the performance of BAKS. In real neural data, the number of spikes (intensity) and the temporal fluctuation of spikes (frequency) may change slowly and rapidly. Therefore, we varied the values of intensity and frequency parameters as described in Table 1 (Testing 2). Using dataset testing 2, we performed 100 repetitions of single trial firing rate estimation from all methods. The statistical summaries of MISE comparison across all methods for the cases of IG and IIG models are shown in Fig 6 and S1 Fig, respectively. From a total of 24 cases (2 models, 3 rate functions and 4 variations of intensity and frequency), BAKS outperforms other competing methods in 16 cases (66.67%) as shown in Fig 6 and S1 Fig. On average, BAKS performs better than other methods. Large performance improvements by BAKS are achieved in the cases of chirp and sawtooth rate functions with high frequency. This suggests that BAKS is more effective at estimating a continuous or discontinuous rate function with rapidly changing spike dynamic. On the other hand, BAKS exhibits poor performance in the case of sine rate function with low frequency. This suggests that BAKS is not suitable for estimating a continuous rate function with slowly changing spike dynamic.

### Comparison under different values of shape parameter (*γ*)

**Fig 7:**
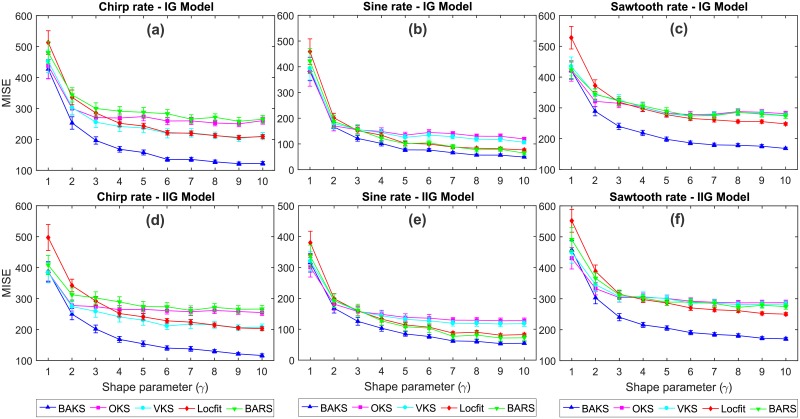
MISE comparison under various values of shape parameter (*γ*). (a)-(c) MISE comparison for chirp, sine, and sawtooth rate functions, respectively, from IG model. (d)-(f) MISE comparison for chirp, sine, and sawtooth rate functions, respectively, from IIG model. Vertical bars represent the 95% confidence intervals.

The flexibility of BAKS when the assumption of ISI shape deviates from that of used during the tuning phase is addressed in this section. Using dataset testing 3 (*γ* = {1, 2, ⋯ 10}), we performed 100 repetitions of single trial firing rate estimation. From a total of 60 cases (2 models, 3 rate functions and 10 variations of *γ* values), BAKS shows superior performance over the other methods in 55 cases (91.67%) as shown in Fig 7. In the remaining cases, BAKS results in comparable performance to OKS and VKS which perform good under inhomogeneous Poisson model (corresponds to *γ* = 1). We also investigated the performance of BAKS when *γ* < 1 which corresponds to bursting activity. We performed firing rate estimations using synthetic data generated with *γ* = {0.25, 0.5, 0.75}. The results show the superiority of BAKS to other methods in all 18 scenarios (2 models, 3 rate functions, 3 variations of *γ* values) as depicted in S2 Fig. These results demonstrate the flexibility of BAKS in estimating the underlying rates under various ISI characteristics of spike train models.

### Comparison under different numbers of trials

**Fig 8:**
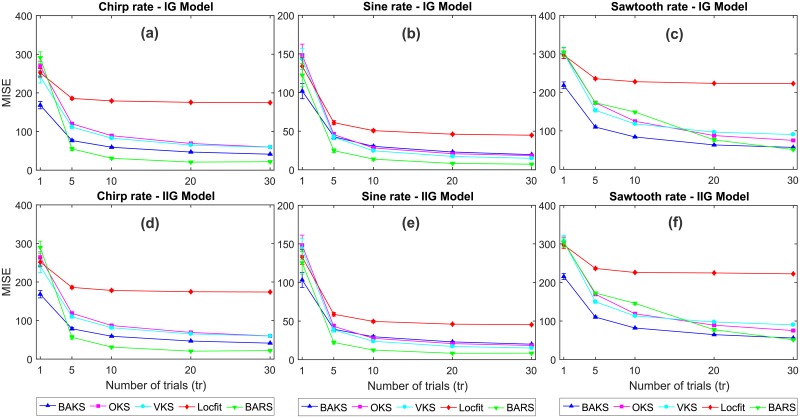
MISE comparison under different numbers of trials. (a)-(c) MISE comparison for chirp, sine and sawtooth rate functions, respectively, for IG model. (d)-(f) MISE comparison for chirp, sine and sawtooth rate functions, respectively, for IG model. Vertical bars (not clearly seen for *tr* ≥ 5) represent the 95% confidence intervals.

In offline analyses, the firing rate is typically estimated using spike trains from many similar trials aggregated into a single, compact, spike train. To study the effect of increasing number of trials, we assessed the performance of BAKS under different numbers of trials (*tr* = {5, 10, 20, 30}) using dataset testing 4. We performed firing rate estimation using all methods, each for 100 times. MISE comparison for these multi-trial cases is depicted in Fig 8. The increasing number of trials improves the performance of all methods as indicated by the decreasing MISE values. However, with the increasing number of trials, the rate of improvement declines as the MISE values reach their convergences. BAKS on average performs better than other methods except BARS. Compared to Locfit, BAKS always shows significantly better performance in all multi-trial cases. In comparison to OKS and VKS, BAKS shows worse performance only in the cases of sine rate functions for *tr* ≥ 10 (Fig 8b and 8e). However, compared to BARS, BAKS shows inferior performance in most multi-trial cases with the exception of sawtooth rate cases (*tr* < 30) as can be seen in Fig 8c and 8f. These overall results suggest that BAKS perform well on multi-trial cases with moderate number of spikes. Nevertheless, when it comes to multi-trial cases with large number of spikes, BARS always performs the best among others.

### Comparison under different underlying rate functions

**Fig 9:**
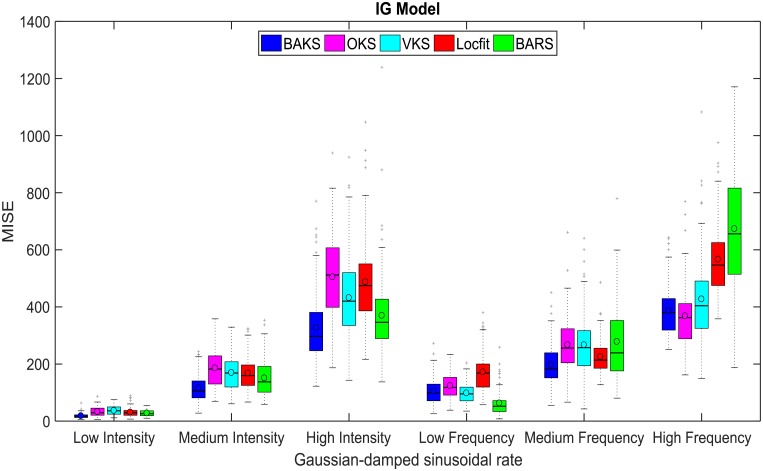
MISE comparison under different underlying rate functions for IG model. In each boxplot, black lines show the medians; black circles indicate the means; colored solid boxes represent interquartile ranges; whiskers extend 1.5× from upper and lower quartiles; gray crosses represent outliers.

In practice, the true underlying rate function that generates the spiking data is unknown. There is infinite spaces of rate function that underlie the spiking generation. During the tuning phase, we used 3 underlying rate functions (chirp, sine and sawtooth) to tune the parameter of BAKS that minimizes the MISE. Next, we studied the impact of different underlying rate function along with its intensity and frequency variations to the performance of BAKS. We performed 100 repetitions of firing rate estimation using dataset testing 5 which corresponds to single trial spike trains generated from Gaussian-damped sinusoidal rate function as in Eq (26). This rate function is chosen as another representation of continuous rate functions with changing spike dynamic. The performance comparisons across all methods using this dataset for IG and IIG models are plotted in Fig 9 and S3 Fig, respectively. From a total of 12 cases, BAKS outperforms all other methods in 8 cases (66.67%).

We also evaluated the performance of BAKS using synthetic data generated from square rate function which represents discontinuous rate functions with changing spike dynamic. The choice of square rate function is inspired by other studies [12, 42]. We performed firing rate estimations using synthetic data generated from IG model with square rate function. We varied the values of frequency and intensity of the square rate function. From a total of 5 cases, BAKS always shows superior performance than other methods as depicted in S5 Fig. These overall results demonstrate the reliability of BAKS even when the underlying rate functions depart from that of used during the tuning phase.

**Fig 10:**
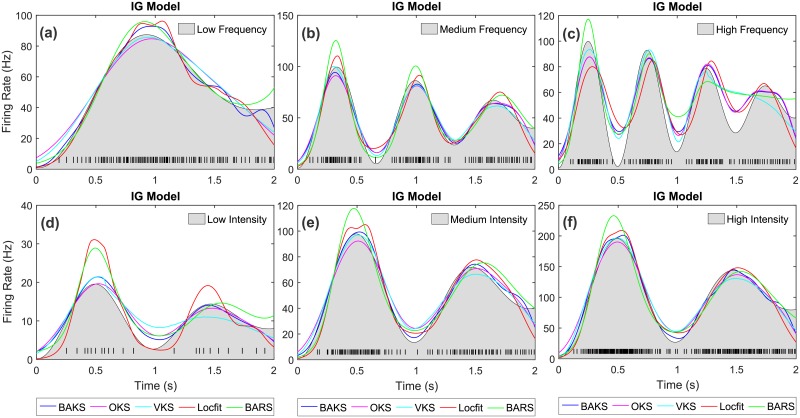
Firing rate estimate comparison for IG model with Gaussian-damped sinusoidal rate function. (a)-(c) Firing rate estimates for the cases of low, medium and high frequency, respectively. (d)-(f) Firing rate estimates for the cases of low, medium and high intensity, respectively. Black lines with gray-shaded regions indicate the underlying rate functions. Black raster in the bottom of each plot represents a spike train generated from the associated underlying rate function.

Examples of estimated firing rates from all methods for IG and IIG models with Gaussian-damped sinusoidal rate function are shown in Fig 10 and S4 Fig, respectively, whereas examples of estimated firing rates for IG model with square rate function are depicted in S6 Fig.

### Comparison of computational complexity

Computational complexity affects how fast the computation of each method is completed. A computationally fast method allows significant reduction in data analysis process when working with large number of iterations. Furthermore, fast computation of firing rate is necessary in online spike-based BMI experiments to generate real-time feedback to the subject. This section describes and compares the computational complexity of each method.

Kernel smoothing technique has the advantage of being relatively simple and computationally fast. This is especially the case when the bandwidth is fixed throughout the observation interval such as in OKS. OKS uses binned spike counts within certain bin intervals (centered at estimation times) and kernel function with fixed bandwidth to estimate firing rate. The firing rate computation is performed by convolving the binned spike counts with the kernel function. OKS incorporates a Fast Fourier Transform (FFT) for computing the convolution to further reduce the computation time. In OKS, the bandwidth is selected by minimizing mean integrated squared error (MISE) function over the whole duration [12]. An extension to OKS is VKS, which incorporates a variable bandwidth. This variable bandwidth is computed by minimizing local MISE which requires large number of iterations with varying local interval. This iterative process makes VKS significantly more complex than OKS. Unlike OKS and VKS, Locfit uses a polynomial to fit log-rate function by maximizing a local likelihood function. Locfit has relatively low complexity because it uses a fixed bandwidth selected in manual fashion; it does not employ an automatic selection of bandwidth. In this study, the bandwidth (in term of nearest neighbor) of Locfit was set to 0.4, meaning that the computation involves 40% of the total data within the whole duration.

Our proposed method, BAKS, even though incorporating an automatic selection of adaptive bandwidth, it still offers relatively low computational complexity. This advantage arises from the simple kernel smoothing technique with proper choice of prior distribution and kernel function which leads to closed-form expression of posterior bandwidth. This in turn simplifies the computation process of determining the adaptive bandwidth. This type of convenient closed-form expression cannot be obtained in the case of BARS; thus, a numerical approximation technique is required. BARS uses an iterative procedure involving computationally expensive Markov chain Monte Carlo (MCMC) technique and Bayes information criterion (BIC) to find the optimal smoothing parameters (i.e. number and location of knots). This process takes relatively long computation to yield “converged” results.

The computational complexity among these methods can be indicated by the time required for completing the firing rate estimation in computer simulation (i.e. computational runtime). Since this runtime comparison is impacted by the code implementation of each method, this should be viewed as an estimation of the real computational complexity of each method. The code for BAKS can be downloaded from https://github.com/nurahmadi/BAKS. The OKS and VKS codes can be downloaded from http://www.neuralengine.org/res/kernel.html. The Locfit code is available through http://chronux.org/; while the BARS code is available through http://lib.stat.cmu.edu/~kass/bars/bars.html. All the program codes were written and run in Matlab R2016b software (The Mathworks Inc., Natick, MA) installed on Windows 7 64-bit PC with 8 Intel cores i7-4790 @3.6 GHz and RAM 16 GB. This comparison uses 100 repetitions of single trial synthetic spike train data (2s duration) generated by IG model with chirp rate function.

As shown in Table 2, Locfit and OKS are the two fastest methods; both methods complete the computation within the order of few milliseconds. VKS requires around 2 order of magnitude longer time than both Locfit and OKS, whereas BARS requires the longest time (in the order of seconds). In BARS parameter setting, we set burn-in iterations to 500 and sample iterations to 5000. The BARS runtime can be reduced by setting the burn-in and sample iterations to smaller values. However, this may result in decreasing accuracy as the trade-off. Therefore, these parameters should be carefully set to find good trade-off between accuracy and runtime. Wallstrom et al. suggested that the default values for burn-in and sample iterations are 500 and 2000, respectively [43].

**Table 2:** Average runtime comparison of BAKS with other methods (in second).

Single trial case	BAKS	OKS	VKS	Locfit	BARS
Low intensity	0.0016	0.0017	0.2340	0.0012	4.8438
Medium intensity	0.0062	0.0018	0.3306	0.0012	4.6563
High intentisy	0.0126	0.0017	0.3306	0.0014	4.5527

The runtime performance of BAKS is significantly better than that of both VKS and BARS, but worse than that of Locfit and OKS. Table 2 shows that BAKS runtime is influenced by the intensity of the underlying rate function (i.e. number of spikes), whereas other methods’ runtime is relatively consistent. This is because other methods incorporate binning procedure for the spiking data prior to their core computation. This makes the number of input data fed to the core computation always uniform regardless of the number spikes within the observation interval. This is not the case for BAKS. Our current BAKS code is a straightforward implementation of the formula described in section. In this study, we have not considered the efficient implementation of BAKS. It is important to note, as neurons have a property of refractory period, the number of spikes within observation interval is limited. This guarantees that under single trial cases, even with current implementation code, the runtime performance of BAKS will only decrease up to certain bound.

### Application to real neural data

In this section, we apply our proposed method for estimating the neuronal firing rate from real data obtained from two public neural databases, which are database for reaching experiments and models (DREAM) and neural signal archive (NSA). The DREAM and NSA databases can be accessed from http://crcns.org/ and http://www.neuralsignal.org/, respectively. In the DREAM database, we used Flint_2012 dataset that were recorded from primary motor cortex area (M1) of monkey’s brain when the subject was performing center-out reaching task. Single unit spikes were obtained by using thresholding and offline sorting technique. More detailed information on the recording tools and experimental setup can be found in [44]. In the NSA database, we used nsa2004.1 dataset recorded from visual cortex (MT/V5) area when random dot stimuli was being presented to a monkey [45]. The detailed electrophysiological recording is given in [46].

Unlike the synthetic data in which the true underlying rate function is known (i.e. ground truth), in the case of real neural data, we do not have access to the ground truth. Therefore, the ground truth underlying rate in real neural data was estimated by averaging and smoothing the spike counts across many similar trials. This procedure is similar to the work of Cunningham et al. [27]. We assume that neurons respond similarly upon given same tasks/stimuli, e.g. moving hand to the same target direction or observing the same visual stimuli. These similar trials were selected such that each trial contains greater or equal to 50 spikes/s within observation interval (1s for the Flint_2012 dataset, 2s for the nsa2004.1 dataset). This limited number of spikes was taken on the assumption that neurons likely fire more spikes when performing tasks or receiving stimuli. In this work, we considered only neurons that satisfy this criterion in more than 30 trials in order to obtain sufficiently small error as we observed in multi-trial synthetic data (Fig 8). To this end, we obtained 47 (4) subdatasets with total trial of 1791 (134) for the Flint_2012 (nsa2004.1) dataset. In the Flint_2012 dataset, we aligned the spiking responses over same-direction reaching tasks to the time when the monkey started the actual hand movement (indicated by cursor movement). To make the observation interval the same from inherently different trial duration for each trial, we used on average 200ms before and 800ms after the movement (total duration of 1s). In the nsa2004.1 dataset, the spiking responses were aligned to the time when random moving dot stimuli was firstly presented to the monkey. In this type of experiment, the trial duration was fixed to 2s.

To estimate the ground truth underlying rate, we employed trial averaging and smoothing processes. Trial averaging process was done by superimposing the spike trains from single neuron and averaging the spike counts within predefined bin interval (10ms) across all similar trials. This trial averaging process still results in a coarse or jagged estimate. Therefore, to get a smooth estimate of the underlying rate, a smoothing process is required. The smoothing process was performed by using BARS. BARS was selected because, based on the synthetic data, it demonstrates the most superior performance in multi-trial cases with a large number of trials (see Fig 8). In these multi-trial cases where there exists spike train variability across trials, we increased the sample iteration to 30,000 and burn-in samples to 5,000 to ensure the convergence of BARS results. One of the advantages of BARS is that it provides the output estimate along with its credible interval. In this work, we used 95% credible interval. When computing MISE, we took into account this uncertainty. Before squaring and integrating across observation interval, we normalized the error between ground truth underlying rate obtained from multi-trials (act as a reference) and single-trial estimated firing rate from method of interest by dividing it with upper or lower credible interval. The upper (lower) interval was used when the estimated firing rate is larger (smaller) than the reference. By doing so, we impose more (less) weight when the credible interval is smaller (larger) to adjust the uncertainty brought by the BARS estimation. We call this normalized MISE as a weighted MISE (WMISE) and formulate it as follows,

$$\begin{array}{c} \text{WMISE}\approx \Delta t\sum E{\left[\frac{\hat{\lambda }\left(t\right)-\lambda \left(t\right)}{C\left(t\right)}\right]}^{2} \end{array}$$ (27)

where *C*(*t*) is set to the upper credible interval when $\hat{\lambda }\left(t\right)\geq \lambda \left(t\right)$ and the lower credible interval when $\hat{\lambda }\left(t\right)<\lambda \left(t\right)$. These upper and lower credible intervals calculated by BARS are not uniform.

**Fig 11:**
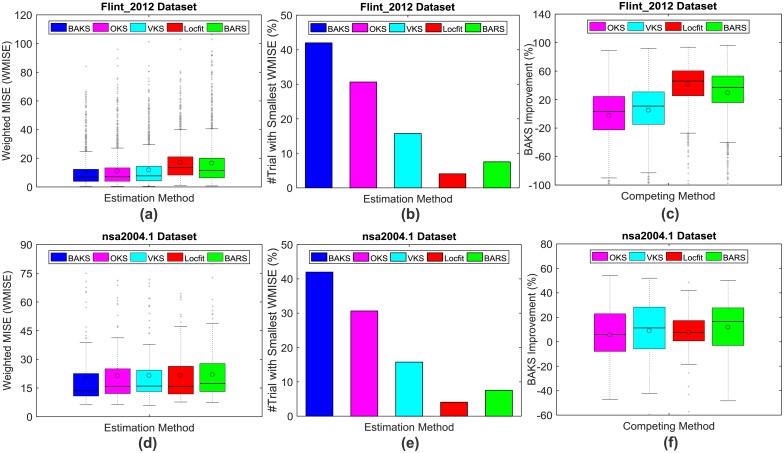
WMISE comparison across all firing rate estimation methods using real neural data. (a) Average WMISE comparison across all trials in Flint_2012 dataset. (b) Number of times (in %) BAKS outperforms other methods in Flint_2012 datasets. (c) Single trial performance improvement made by BAKS over competing methods in Flint_2012 datasets. (d)-(f) Similar to that of (a)-(c) but with nsa2004.1 dataset. In each boxplot, black lines show the medians; black circles indicate the means; colored solid boxes represent interquartile ranges; whiskers extend 1.5× from upper and lower quartiles; gray crosses represent outliers.

We examined the performance comparison across methods under WMISE function. Based on WMISE function, we derived three different metrics for performing the comparison. First, we investigated the WMISE performance across total number of trials. Based on 1791 single-trial firing rate estimation from 47 subdatasets in the Flint_2012 dataset, BAKS produces the smallest WMISE mean (10.34) and median (6.85) as shown in Fig 11a. BAKS also produces the best performance (mean = 19.77 and median = 13.56) in the case of nsa2004.1 dataset with a total of 134 trials from 4 subdatasets (Fig 11d). Second, we measured the number of times (in %) one method outperforms all the other methods. In both Flint_2012 and nsa2004.1 datasets, BAKS more frequently (41.99% and 42.54% respectively) outperforms the other methods as depicted in Fig 11b and 11e. In these cases, OKS comes as the second best method with 30.65% and 17.16%. Third, we assessed the improvement (WMISE decrease in %) per trial made by BAKS against other methods. Fig 11c and 11f describe the statistical summary of BAKS performance compared to competing methods for the Flint_2012 and nsa2004.1 datasets, respectively. A positive (negative) value means that the BAKS method outperforms (is outperformed by) the others. As described in Fig 11c, the performance of BAKS is better than other methods except OKS (see magenta boxplot; mean = -2.58% and median = 3.20%). This seems to contradict the results when using the first and second metrics, in which BAKS outperforms all other methods. To investigate this contradictory, we analyzed the performance of BAKS against OKS across 1791 trials. It turns out that although BAKS more frequently outperforms OKS, in a few trials, the magnitude of improvement made by OKS against BAKS is significantly large. However, in the nsa2004.1 dataset, BAKS is able to outperform all other methods (Fig 11f). These results are in agreement with the two other metrics and the results obtained from the synthetic datasets. As a summary, BAKS on average performs good compared to other methods in both real and synthetic datasets.

**Fig 12:**
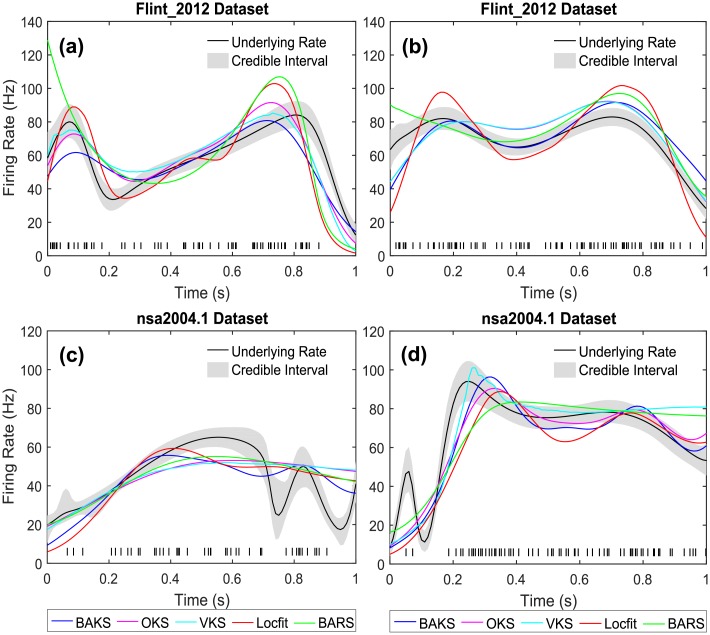
Firing rate estimates comparison for real neural data. (a) Firing rate estimates from ‘N184’ of Flint_2012 dataset (b) Firing rate estimates from ‘E164’ of Flint_2012 dataset (c) Firing rate estimates from ‘j032_25.6’ of nsa2004.1 dataset. (d) Firing rate estimates from ‘j032_51.2’ of nsa2004.1 dataset. Black lines and gray-shaded regions indicate the ‘true’ underlying rates and their 95% credible intervals, respectively. Black raster in the bottom of each plot represents a single spike train generated from the associated underlying rate.

Some examples of single-trial firing rate estimations from all methods for both datasets are shown in Fig 12. Fig 12a and 12b show the firing rate estimates from ‘N184’ and ‘E164’ cases in the Flint_2012 dataset. Fig 12c and 12d show the firing rate estimates from ‘j032_25.6’ and ‘j032_51.2’ cases in the nsa2004.1 dataset.

## Discussion

In this study, we propose a new method, referred to as BAKS, for estimating single-trial neuronal firing rate. BAKS employs a kernel smoothing technique with adaptive bandwidth. This differs from other kernel-based firing rate estimation methods in that its bandwidth parameter is adaptively determined by an empirical Bayesian approach. BAKS has been developed with the motivation to estimate firing rate from single spike train generated from underlying rate function that dynamically changes over observed duration (i.e. non-stationary).

We tune the parameter of BAKS using synthetic spike train data stochastically sampled from 3 rate functions (as representation of non-stationary underlying processes). These rate functions are chirp, sine, and sawtooth expressed in Eqs (23), (24) and (25), respectively. Using this tuned parameter, we evaluate the performance of BAKS using 5 synthetic datasets. These datasets represent various setting and combination of underlying rate functions along with their intensity and frequency variations, ISI shape (parameter *γ*), and number of trials. The performance comparison is measured under MISE function. By extensive simulations, we demonstrate good performance of BAKS compared to two other kernel-based methods (OKS and VKS) and two generalized nonparametric regression methods (Locfit and BARS). On average, BAKS outperforms the other methods in single-trial estimation (smallest MISE) across various settings. The adaptive bandwidth featured in the BAKS can adjust the different spike densities within the observation interval. The results suggest that BAKS is suitable to be used for single-trial analysis of neural data. The flexibility of BAKS has also been tested by using spike train generated from same models with different values of shape parameter (*γ* = {0.25, 0.5, 0.75, 1, 2, 3, ⋯ 10}). These various values represent diverse neuronal activities which include bursting, irregular, and regular spiking. Furthermore, BAKS is also evaluated using synthetic datasets generated from two other underlying rate functions, namely Gaussian-damped sinusoidal rate and square rate functions. Consistent results are obtained despite using these different characteristics of spike trains. BAKS does not assume specific distribution on the spike train, rather it uses appropriately chosen prior distribution on the bandwidth parameter. The prior distribution of the bandwidth is derived from Gamma prior distribution on the precision parameter (inverse of square bandwidth). The precision parameter describes how concentrated observed data are around the means of Gaussian kernel which are set to the spike times. Since these spike times (i.e. sum of ISI) are conveniently modeled with Gamma distribution [15, 27–30], the precision parameter is also assumed to be Gamma distribution. This choice has been shown to yield good performance. On the other hand, all other competing methods (OKS, VKS, Locfit, and BARS) use a Poisson assumption [12–14], which is less likely for the case of single-trial spike train; neurons have certain properties (e.g. refractory and bursting) that cannot be described by the Poisson model [15, 16]. Numerous works have shown the inadequacy of the Poisson model and proposed other more biophysically plausible models (e.g. IG and IIG) [6, 15, 27–30, 36]. The deviation from Poisson assumption under single-trial cases may lead OKS, VKS, Locfit and BARS to poor performance [12, 17].

We also compare the performance of BAKS method under multi-trial cases to study the implication of increasing number of spikes within the same duration. Some topics of interest in neuroscience use multi-trial spike train to obtain the firing rate. The results show that all methods produce similar trend; the increasing number of trials up to a certain value (threshold) improves the performance. However, as the number of trial increases, the rate of improvement of each method and the threshold value differ from each other. BARS produces the most significant performance improvement with the increasing number of trials. This significant improvement of BARS is driven by two factors. First, BARS is built on the assumption that spike counts within the bin intervals follow a Poisson distribution. This assumption is suitable for multi-trial cases since superimposed spike train across trials approximate a Poison process [12, 16]. Second, BARS employs a fully Bayesian approach and locality heuristic procedure to update prior distribution of parameter (knots configuration) [14]. With the increasing number of trials (i.e. number of spikes), BARS at the cost of high computational complexity can effectively find the optimal knots configuration regardless of the prior. Nevertheless, in comparison to OKS, VKS, and Locfit methods, BAKS still yields good performance. The overall results suggest that BAKS is good at estimating firing rate from a low to moderate number of spikes (represented by single or few trials) from non-stationary underlying rate functions. BAKS performs even significantly better in the cases of sawtooth and square rate functions which indicates its suitability for estimating firing rate from discontinuous underlying rate functions.

After validation using synthetic data, BAKS is also tested using real neural data recorded from motor and visual cortex of non-human primate (NHP). The motor neural data (Flint_2012) is associated with center-out reaching tasks, whereas the visual neural data (nsa2004.1) is associated with moving random-dot visual stimuli. Measuring the performance in real neural data is a challenging due to unknown underlying rate. Hence, the underlying rate is estimated by using multi-trial cases on the assumption that neurons respond similarly upon given same tasks/stimuli. This procedure is similar to Cunningham’s work [27]. However, our procedure differs from [27] in that we use BARS instead of PSTH for smoothing process. In practice, neuronal response may considerably differ across similar trials. To minimize large variation in the spike trains, subsets from two datasets (Flint_2012 and nsa2004.1) are selected with constraints explained in previous section. BARS is chosen to obtain a smooth estimate of the ground truth underlying rate as it provides the smallest MISE in multi-trial cases of synthetic data. To account for the estimation uncertainty produced by BARS, weighted MISE (WMISE) is used for comparison. From a total of 6 cases (3 metrics and 2 real datasets), BAKS outperforms all other methods in 5 cases. The overall results show that, on average, BAKS yields good performance compared to all other competing methods. This is in good agreement with the results obtained from single-trial synthetic data, which further demonstrates the effectiveness of the proposed method in estimating single-trial neuronal firing rate.

In BAKS, the selection of prior parameter (*α*) value is crucial since it will impact the performance of the firing rate estimation. In this study, the value of *α* is tuned by minimizing MISE on synthetic dataset during training phase. This dataset is generated from three rate functions (chirp, sine, sawtooth) with medium intensity and frequency. During testing phase, we use datasets with various settings to evaluate the robustness of BAKS with this tuned parameter (*α*_*t*_ = 4). According to Eq (13), the mean of prior bandwidth is inversely proportional to the value of *α*. In the cases of low frequency rate functions with the same intensity as of during the tuning phase, the spike density tends to decrease, which in turn requires larger bandwidth. To adapt to this, the value of *α* that minimizes MISE tends to become smaller. Since we use a fixed *α*_*t*_, this results in poor performance of BAKS. On the other hand, in the cases of high frequency, although there is performance degradation, BAKS still shows better performance compared to all other methods. This may be caused by relatively small performance degradation resulted from the use of *α*_*t*_ as opposed to larger value of *α* (for adapting to higher spike density). As can be seen in (Fig 3), the performance difference from the use of *α*_*t*_ = 4 to the right (larger value of *α*) is smaller than to the left (smaller value of *α*). In other cases related with different values of intensity (the frequency is kept same), the performance of BAKS is consistently better among other methods. The change of intensity (thus the associated bandwidth) can be partially compensated by the change in scale parameter (*β*) which is set to a function proportional to the number of spikes. This indicates that the performance of BAKS is less (more) sensitive to *α*_*t*_ value when there is low (high) variation of frequency (i.e. temporal fluctuation) of the underlying rate function.

In practice, the value of *α* parameter of BAKS can be tuned using synthetic datasets generated from spike train model whose parameters derived from statistical summary of real datasets of interest. By analyzing repeated trial spike trains on real neural datasets with the same tasks/stimuli, we can estimate important underlying parameters such as temporal fluctuation, mean and dynamic range of intensity, ISI characteristic, and shape of the rate function. These information can then be fed to the spike train model to generate synthetic datasets for tuning purpose. Using the same principle of minimizing MISE, we can tune *α* and use this tuned value for firing rate estimation during testing phase.

BAKS offers simplicity as standard kernel-based method does, yet is effective in grasping sudden and slow changes of firing rate in different regions within the observation interval. Unlike BARS which is computationally demanding, BAKS is relatively fast owing to an analytical expression of bandwidth posterior density. This analytical expression leads to the adaptive bandwidth determined in an exact way (not numerical approximation), which reduces the computational complexity. With good performance and relatively low complexity, BAKS is suitable to be used for research that require single-trial firing rate estimation. For example, understanding the encoding mechanism of neurons in cognitive-related tasks and decoding task parameter in brain-machine interface (BMI) applications. As a summary, the comparison of BAKS with other methods is given in Table 3.

**Table 3:** Comparison summary of BAKS with other methods.

	BAKS	OKS	VKS	Locfit	BARS
Adaptability to underlying dynamics	✓	−	✓	−	✓
Bayesian/probabilistic approach	✓	−	−	−	✓
Automatic selection of smoothing parameter	✓	✓	✓	−	✓
Single trial (low to moderate number of spikes)	◇ ◇ ◇	◇◇	◇◇	◇◇	◇◇
Multi trials (large number of spikes)	◇◇	◇◇	◇◇	◇	◇ ◇ ◇
Computational complexity (runtime)	◇ ◇ ◇	◇ ◇ ◇	◇◇	◇ ◇ ◇	◇

More diamonds mark (◇) indicates better (desirable) property.

## Conclusion

We have presented a simple yet accurate method, referred to as BAKS, for estimating single-trial neuronal firing rate based on a kernel smoothing technique with adaptive bandwidth. The key idea of BAKS is to consider the bandwidth parameter as a random variable under an empirical Bayesian framework. By using Bayes’ theorem with proper choice of kernel and prior distribution functions, the bandwidth can be adaptively determined in an exact and quick way. Extensive evaluations on both synthetic and real neural data show that BAKS yields good performance compared to other competing methods. This suggests that BAKS has the potential to improve single-trial analysis in neuroscience studies and decoding performance of spike-based brain-machine interfaces (BMIs).

## Appendix

### Appendix 1. Posterior distribution of bandwidth

In this appendix, we derive a closed-form expression of the posterior density of bandwidth as given in Eq (9). According to Bayes’ theorem, the posterior density is formulated as:

$$\begin{array}{c} \pi \left(h\left(t\right)|\rho \left(t\right)\right)=\frac{\hat{f}\left(\rho \left(t\right)|h\left(t\right)\right)\pi \left(h\left(t\right)\right)}{\int \hat{f}\left(\rho \left(t\right)|h\left(t\right)\right)\pi \left(h\left(t\right)\right)dh\left(t\right)} \end{array}$$ (28)

Using likelihood function as in Eq (7) and prior distribution of bandwidth as in Eq (6), we can obtain:

$$\begin{array}{c} \pi \left(h\left(t\right)|\rho \left(t\right)\right)=\frac{\frac{1}{n}\sum_{i=1}^{n}K_{h(t)}\left(t-t_{i}\right)\frac{2h{\left(t\right)}^{-2\alpha -1}}{\Gamma \left(\alpha \right)\beta^{\alpha }}\exp\{-\frac{1}{\beta h{\left(t\right)}^{2}}\}}{\int \frac{1}{n}\sum_{i=1}^{n}K_{h(t)}\left(t-t_{i}\right)\frac{2h{\left(t\right)}^{-2\alpha -1}}{\Gamma \left(\alpha \right)\beta^{\alpha }}\exp\{-\frac{1}{\beta h{\left(t\right)}^{2}}\}dh\left(t\right)} \end{array}$$ (29)

By substituting a Gaussian kernel into the likelihood function and removing the same constants in both numerator and denominator, Eq (29) then becomes:

$$\begin{array}{c} \pi \left(h\left(t\right)|\rho \left(t\right)\right)=\frac{\sum_{i=1}^{n}h{\left(t\right)}^{2\alpha -2}\exp\{-\frac{1}{h{\left(t\right)}^{2}}[\frac{{\left(t-t_{i}\right)}^{2}}{2}+\frac{1}{\beta }]\}}{\int \sum_{i=1}^{n}h{\left(t\right)}^{2\alpha -2}\exp\{-\frac{1}{h{\left(t\right)}^{2}}[\frac{{\left(t-t_{i}\right)}^{2}}{2}+\frac{1}{\beta }]\}dh\left(t\right)} \end{array}$$ (30)

Let now consider the denominator of Eqs (28) and (30), which we can rewrite as:

$$\begin{array}{c} \int \hat{f}\left(\rho \left(t\right)|h\left(t\right)\right)\pi \left(h\left(t\right)\right)dh\left(t\right)=\int \sum_{i=1}^{n}h{\left(t\right)}^{2\alpha -2}\exp\{-\frac{1}{h{\left(t\right)}^{2}}[\frac{{\left(t-t_{i}\right)}^{2}}{2}+\frac{1}{\beta }]\}dh\left(t\right) \end{array}$$ (31)

To simplify the calculation, let us define variables as follows:

$$\begin{array}{c} \sigma \left(t\right)=\frac{1}{h{\left(t\right)}^{2}},\;h\left(t\right)=\sigma {\left(t\right)}^{-\frac{1}{2}},\;dh\left(t\right)=-\frac{1}{2}\sigma {\left(t\right)}^{-\frac{3}{2}}d\sigma \left(t\right) \end{array}$$ (32)

$$\begin{array}{c} \theta \left(t\right)={\left[\frac{{\left(t-t_{i}\right)}^{2}}{2}+\frac{1}{\beta }\right]}^{-1} \end{array}$$ (33)

By substituting Eqs (32) and (33) into Eq (31), the integral function can be represented as:

$$\begin{array}{c} \int \hat{f}\left(\rho \left(t\right)|h\left(t\right)\right)\pi \left(h\left(t\right)\right)dh\left(t\right)=\frac{1}{2}\sum_{i=1}^{n}\int \sigma {\left(t\right)}^{\alpha -\frac{1}{2}}\exp\{-\frac{\sigma (t)}{\theta (t)}\}d\sigma \left(t\right) \end{array}$$ (34)

Eq (34) can be simplified so that the integral part forms Gamma probability density as follows:

$$\begin{array}{c} \begin{array}{cc} \int \hat{f}\left(\rho \left(t\right)|h\left(t\right)\right)\pi \left(h\left(t\right)\right)dh\left(t\right) & =\frac{1}{2}\sum_{i=1}^{n}\Gamma \left(\alpha +\frac{1}{2}\right)\theta {\left(t\right)}^{\left(\alpha +\frac{1}{2}\right)}\\ & \;\times \int \frac{\sigma {\left(t\right)}^{(\alpha +\frac{1}{2})-1}}{\Gamma \left(\alpha +\frac{1}{2}\right)\theta {\left(t\right)}^{\left(\alpha +\frac{1}{2}\right)}}\exp\{-\frac{\sigma (t)}{\theta (t)}\}d\sigma \left(t\right) \end{array} \end{array}$$ (35)

Since the integration of Gamma probability density function is equal to 1,

$$\begin{array}{c} \int \frac{\sigma {\left(t\right)}^{(\alpha +\frac{1}{2})-1}}{\Gamma \left(\alpha +\frac{1}{2}\right)\theta {\left(t\right)}^{\left(\alpha +\frac{1}{2}\right)}}\exp\{-\frac{\sigma (t)}{\theta (t)}\}d\sigma \left(t\right)=1 \end{array}$$ (36)

Eq 35 can then be analytically expressed as:

$$\begin{array}{ll} \int \hat{f}(\rho \left(t\right)|h\left(t\right))\pi \left(h\left(t\right)\right)dh\left(t\right) & =\frac{1}{2}\sum_{i=1}^{n}\Gamma \left(\alpha +\frac{1}{2}\right)\theta {\left(t\right)}^{\left(\alpha +\frac{1}{2}\right)}\\ & =\frac{1}{2}\Gamma \left(\alpha +\frac{1}{2}\right)\sum_{i=1}^{n}{\left[\frac{{\left(t-t_{i}\right)}^{2}}{2}+\frac{1}{\beta }\right]}^{\left(-\alpha -\frac{1}{2}\right)} \end{array}$$ (37)

Finally, by substituting Eq (37) back to the original equation of posterior density of bandwidth in Eq (28), we can obtain the closed-form solution as in Eq (9):

$$\begin{array}{c} \pi \left(h\left(t\right)|\rho \left(t\right)\right)=\frac{\sum_{i=1}^{n}h{\left(t\right)}^{-2\alpha -2}\exp\{-\frac{1}{h{\left(t\right)}^{2}}[\frac{{\left(t-t_{i}\right)}^{2}}{2}+\frac{1}{\beta }]\}}{\frac{1}{2}\Gamma \left(\alpha +\frac{1}{2}\right)\sum_{i=1}^{n}{\left[\frac{{\left(t-t_{i}\right)}^{2}}{2}+\frac{1}{\beta }\right]}^{\left(-\alpha -\frac{1}{2}\right)}} \end{array}$$ (38)

### Appendix 2. Adaptive bandwidth estimate

Under squared error loss function, the adaptive bandwidth can be estimated by using the posterior mean as given by:

$$\begin{array}{c} \hat{h}\left(t\right)=\int h\left(t\right)\pi \left(h\left(t\right)|\rho \left(t\right)\right)dh\left(t\right) \end{array}$$ (39)

By substituting Eq (38) into Eq (39), we can obtain:

$$\begin{array}{c} \hat{h}\left(t\right)=\frac{\int \sum_{i=1}^{n}h{\left(t\right)}^{-2\alpha -1}\exp\{-\frac{1}{h{\left(t\right)}^{2}}[\frac{{\left(t-t_{i}\right)}^{2}}{2}+\frac{1}{\beta }]\}dh\left(t\right)}{\frac{1}{2}\Gamma \left(\alpha +\frac{1}{2}\right)\sum_{i=1}^{n}{\left[\frac{{\left(t-t_{i}\right)}^{2}}{2}+\frac{1}{\beta }\right]}^{\left(-\alpha -\frac{1}{2}\right)}} \end{array}$$ (40)

Similar to the derivation procedure for the posterior distribution of bandwidth (Appendix 1), by the change-of-variables rule using Eqs (32), (33) and (40) can be written as:

$$\begin{array}{c} \hat{h}\left(t\right)=\frac{\frac{1}{2}\sum_{i=1}^{n}\int \sigma {\left(t\right)}^{\alpha -1}\exp\{-\frac{\sigma (t)}{\theta (t)}\}d\sigma \left(t\right)}{\frac{1}{2}\Gamma \left(\alpha +\frac{1}{2}\right)\sum_{i=1}^{n}{\left[\frac{{\left(t-t_{i}\right)}^{2}}{2}+\frac{1}{\beta }\right]}^{\left(-\alpha -\frac{1}{2}\right)}} \end{array}$$ (41)

By modifying the integral part of numerator to be an integration of Gamma probability density function (which is equal to 1) as in Eq (35), we can obtain the final closed-form solution as in Eq (11):

$$\begin{array}{c} \hat{h}\left(t\right)=\frac{\Gamma \left(\alpha \right)\sum_{i=1}^{n}{\left[\frac{{\left(t-t_{i}\right)}^{2}}{2}+\frac{1}{\beta }\right]}^{-\alpha }}{\Gamma \left(\alpha +\frac{1}{2}\right)\sum_{i=1}^{n}{\left[\frac{{\left(t-t_{i}\right)}^{2}}{2}+\frac{1}{\beta }\right]}^{-\alpha -\frac{1}{2}}} \end{array}$$ (42)

## Supporting information

S1 Fig MISE comparison under different values of intensity and frequency for IIG model. (a) MISE comparison for the case of low intensity. (b) MISE comparison for the case of high intensity. (c) MISE comparison for the case of low frequency. (d) MISE comparison for the case of high frequency. In each boxplot, black lines show the medians; black circles indicate the means; colored solid boxes represent interquartile ranges; whiskers extend 1.5× from upper and lower quartiles; gray crosses represent outliers.
(EPS)

S2 Fig MISE comparison under different values of shape parameter (*γ* = {0.25, 0.5, 0.75}). (a)-(c) MISE comparison for chirp, sine, and sawtooth rate functions, respectively, from IG model. (d)-(f) MISE comparison for chirp, sine, and sawtooth rate functions, respectively, from IIG model. Vertical bars represent the 95% confidence intervals.
(EPS)

S3 Fig MISE comparison for IIG model with Gaussian-damped sinusoidal rate function. In each boxplot, black lines show the medians; black circles indicate the means; colored solid boxes represent interquartile ranges; whiskers extend 1.5× from upper and lower quartiles; gray crosses represent outliers.
(EPS)

S4 Fig Firing rate estimate comparison for IIG model with Gaussian-damped sinusoidal rate function. (a)-(c) Firing rate estimates for the cases of low, medium and high frequency, respectively. (d)-(f) Firing rate estimates for the cases of low, medium and high intensity, respectively. Black lines with gray-shaded regions indicate the underlying rate functions. Black raster in the bottom of each plot represents a spike train generated from the associated underlying rate function.
(EPS)

S5 Fig MISE comparison for IG model with square rate function. In each boxplot, black lines show the medians; black circles indicate the means; colored solid boxes represent interquartile ranges; whiskers extend 1.5× from upper and lower quartiles; gray crosses represent outliers.
(EPS)

S6 Fig Firing rate estimate comparison for IG model with square rate function. (a)-(b) Firing rate estimates for the cases of low and high frequency, respectively. (c)-(d) Firing rate estimates for the cases of low and high intensity, respectively. Black lines with gray-shaded regions indicate the underlying rate functions.
(EPS)
